# Stirring does not make populations well mixed

**DOI:** 10.1038/s41598-018-22062-w

**Published:** 2018-03-06

**Authors:** Francisco Herrerías-Azcué, Vicente Pérez-Muñuzuri, Tobias Galla

**Affiliations:** 1 Theoretical Physics, School of Physics and Astronomy, The University of Manchester, M13 9PL Manchester, United Kingdom; 2 Group of Nonlinear Physics, Faculty of Physics, University of Santiago de Compostela, E-15782 Santiago de Compostela, Spain

## Abstract

In evolutionary dynamics, the notion of a ‘well-mixed’ population is usually associated with all-to-all interactions at all times. This assumption simplifies the mathematics of evolutionary processes, and makes analytical solutions possible. At the same time the term ‘well-mixed’ suggests that this situation can be achieved by physically stirring the population. Using simulations of populations in chaotic flows, we show that in most cases this is not true: conventional well-mixed theories do not predict fixation probabilities correctly, regardless of how fast or thorough the stirring is. We propose a new analytical description in the fast-flow limit. This approach is valid for processes with global and local selection, and accurately predicts the suppression of selection as competition becomes more local. It provides a modelling tool for biological or social systems with individuals in motion.

## Introduction

Population dynamics describes the changes of the composition of a group of individuals over time. Broadly speaking, there are two modelling approaches. One involves well-mixed populations, implying an all-to-all interaction. This is contrasted with structured populations, or populations on networks. Mathematically, the interaction network of well-mixed populations is often assumed to be a ‘complete graph’ (see e.g.^1–4^), i.e., a network in which interaction links exist between any two individuals at all times. In the context of epidemics, for example, an infection event can affect any of the susceptible individuals in the population; in evolutionary dynamics, it indicates that competition occurs between all members of the population. This effectively means that there is no spatial structure at all, or at least that interaction is sufficiently long-range that spatial structure is not relevant for the evolutionary process.

Assume we place a population of discrete individuals in a container and stir the system. In an experimental situation this could be a bacterial population in a stirred-tank reactor, for example, or swimmers who move on their own accord^5–8^. One would naturally think that a well-mixed system can be obtained in this way, provided the stirring is sufficiently thorough and that one waits long enough. We use computer simulations and analytical theory to study this scenario. We take a finite population and immerse it in different chaotic flows to mimic stirring. We focus on the situation in which a single mutant invades this population, and ask when the theory for well-mixed populations quantitatively predicts its chances of success. In the language of population genetics, we study the probability of fixation^9^.

Work has been done studying the fixation probability on fixed random graphs^1,10^ or adaptive networks^11,12^, where links are rewired to benefit individuals. Our model is different in that the changes of the interaction network are solely induced by the flow, and cannot be controlled by the individuals at the nodes of the graph. This is closer to what is studied in^13^. In the present paper, however, we focus on frequency-independent selection, so our results cannot directly be compared to those of^13^.

Our simulations and analysis show that the predictions of the conventional theory for well-mixed populations do not always capture the outcome of evolutionary processes in stirred environments. Its validity seems not to be primarily a question of the nature or speed of the stirring; instead, it is determined by the interaction range and the type of evolutionary process. As a consequence we think the term ‘well-mixed’, which at least suggests external stirring, needs to be used with care.

We present an analytical approach to describe stirred populations, in which we abandon the assumption that the interaction graph is complete at all times. Instead, we rely on a broader definition: a population is well mixed if every pair of individuals interacts with the same probability^14–16^. This does not imply, however, that competition occurs among all individuals at all times. At any one time particles take positions in space and compete within an interaction radius. Any given individual therefore competes only against a subset of individuals in the population. If the population is stirred at sufficiently high rates, and if the flow is such that it ‘mixes’ all parts of the system, the positions of the particles are random at each evolutionary step. The possible interaction partners of a given individual are then effectively sampled uniformly from the entire population, and any two individuals are equally likely to interact. This process can be described analytically, and fixation probabilities can be obtained. In contrast to the conventional theory for well-mixed populations, this method accurately reproduces simulation results for stirred systems.

We show that the conventional theory is only valid for processes in which selection is global, i.e., it occurs between *all* individuals in the population. The method presented here, on the other hand, is also valid for local selection, or a combination of the two. Since the details of the evolutionary dynamics in real-world systems are rarely known with certainty, this flexibility makes our approach relevant for the modelling of experiments where the interacting individuals are in motion.

## Results and Methods

### Mathematical definition of well-mixed populations

It is fair to say that there is a consensus on what constitutes a well-mixed population in mathematical models of evolutionary dynamics. In order to illustrate this, we focus on stochastic dynamics in finite populations, and use a discrete-time process with frequency-independent fitness. In the first instance we choose a so-called death-birth update; this is sufficient to present our results. Other mechanics of evolution (for example birth-death processes) are considered in the Supplemental Material.

The population consists of *N* individuals; we assume that its size is constant over time. Each individual can either be a mutant or a wildtype. The state of the population at any point in time is characterised by the number of mutants, *m*; the number of wildtypes is *N* − *m*. In a traditional well-mixed approach the actual positions of the individuals in space are irrelevant, as everyone can interact with everyone else at all times. There is then nothing else to know about the state of the population. Evolution occurs through combined death-birth events. In each event, one individual is picked at random, regardless of its type, and is removed from the population. One of the remaining individuals is then selected for reproduction and generates an offspring. Selection is based on fitness, and the offspring is of the same species as its parent. We assume frequency-independent selection, and set the fitness of wildtype individuals to one. The invading mutant has fitness *r*, which can be smaller than one (for disadvantageous mutations) or greater than one (for advantageous mutations). For *r* = 1 the process is neutral.

The dynamics proceed through a sequence of death-birth events until the mutants have either gone extinct (*m* = 0), or reached fixation (*m* = *N*). When either has occurred the dynamics stop, as there is only one type of individual left in the population. We focus on the probability, *ϕ*, for a single invading mutant to reach fixation. Using the theory of Markov processes, an explicit mathematical expression can be found for the fixation probability (see e.g.^9,17–19^). The fixation probability depends on the fitness of the mutant species, *r*, and the population size, *N*. For the death-birth process one has^3,20^

1 $$\varphi =c\,\frac{1-{r}^{-1}}{1-{r}^{-cN}},$$

where *c* = (*N* − 1)/*N* for the process described above. We will refer to this as the prediction of the conventional theory for well-mixed populations. If asked how to calculate fixation probabilities in well-mixed populations, most evolutionary theorists would likely point to results such as the one in Eq. (1). The details of the mathematical expression may vary for different processes (for example *c* = 1 if self-replacement is included, or if the selection is global), but they are all derived from the assumption of an all-to-all interaction at all times.

#### Can this be achieved by stirring

Despite the consensus on what constitutes a well-mixed population (mathematically), it is in practice difficult to determine if a particular biological or social system is well mixed. The term is used in the literature without much specificity. Common verbal characterisations include the requirement that ‘all pairs of individuals interact with the same probability’ (see e.g.^14–16,21^), but how precisely this is to be interpreted is often not said. For example, do all individuals have to be able to interact with each other at all times? Or it is sufficient if all pairs interact with equal frequency over time? In most cases no detailed explanation is offered how the complete interaction graph leading to Eq. (1) would arise. The term attributed to this formalism–evolutionary dynamics in ‘well-mixed’ populations–at the very least suggests that this all-to-all interaction can be achieved through some type of external stirring or agitation. It is this assumption that we challenge in this paper.

### Models of stirred populations

In order to model the effects of external stirring we assume that the population is subject to a continuous-time flow, moving the individuals around in space^22–30^. For example, one may imagine a population of bacteria in an aqueous environment, which is being shaken or stirred mechanically^5–8^. We focus on two-dimensional systems; this is sufficient to develop the main points we would like to make.

Each evolutionary death-birth event of the Moran process is executed as follows. First, one individual in the population is chosen at random for removal; each individual with equal probability 1/*N*. Then, its ‘neighbours’ (individuals within interaction range *R*) compete to reproduce and fill the vacancy. This competition is decided by fitness: assuming that *n* wildtypes and *m* mutants compete, the probability that the reproducing individual is a wildtype is *n*/(*n* + *mr*), and the probability that a mutant reproduces is *mr*/(*n* + *mr*). The offspring is created with the same type as the parent, and is placed at the position of the individual that has been removed.

It remains to say how often evolutionary events take place relative to the timescale of the flow. In-line with the literature we will describe this by the so-called Damköhler number, *Da* (see e.g.^30–33^); in the context of our model, the Damköhler number characterises the ratio of the time scales of the flow and the evolutionary process. In our simulations, one evolutionary event occurs every 1/(*NDa*) time units. If *Da* is very large, the flow is slow compared to evolution. The extreme case *Da* → ∞ describes the ‘no-flow’ limit; on the timescale of the evolutionary dynamics, the positions of the individuals are then static. A very small Damköhler number $(\,{\rm{Da}}\ll 1)$ indicates that the flow is fast compared to evolution. If the conventional theory for well-mixed populations is to apply to populations in a flow, then one would expect it to be in this limit.

We performed simulations of the evolutionary process in different chaotic flows. The velocity fields we use have a periodic time dependency. The time units are expressed in units of the period of the flows, which we set to unity throughout. As an initial example, we focus on the so-called periodic parallel-shear flow^34,35^ in two dimensions. In this flow, particles move horizontally during the first half of the period, subject to shear. In the second half, the motion is in vertical direction, again subject to shear. A random phase, drawn every half period, leads to chaotic motion^34,35^. We will discuss further flows below. Details of the flows, including the numerical methods we used to simulate them, are described in the Supplemental Material.

**Figure 1:**
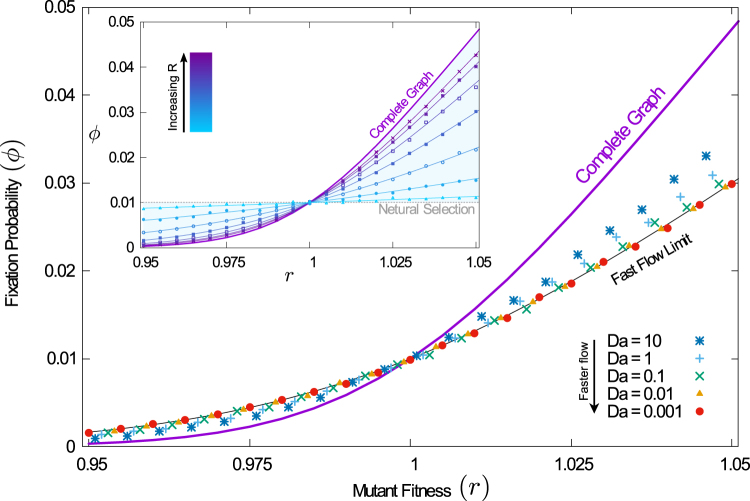
Fixation probability of a single mutant in a population stirred by a chaotic flow. The conventional theory for well-mixed systems [Eq. (1)] is shown as a thick purple line. Markers represent simulation results. In the main panel, these are shown for different Damköhler numbers. Reducing *Da* increases the flow speed relative to the evolutionary process. The thin continuous lines represent results from the analytical approach for fast flows [Eq. (2)]. The inset shows simulations for different interaction radii. Smaller interaction ranges makes selection increasingly more local, and the fixation probability approaches that of neutral selection, 1/*N*, shown for reference (dashed gray line). (Population size *N* = 100; *R* = 0.1 in main panel; Da = 0.1 in the inset; interaction radius varies from *R* = 0.025 to *R* = 0.175 in inset. See Supplemental Material for a description of the (parallel-shear) flow).

Results of the simulations for the parallel shear flow are shown in the main panel of Fig. 1. The thick purple line is the prediction of the conventional theory for well-mixed systems. The markers represent simulation results for different Damköhler numbers. For fixed mutant fitness, the data suggests that the fixation probability approaches a limiting value as *Da* is decreased (the flow made faster). However, this limiting value is not the one predicted by Eq. (1). This indicates that the conventional well-mixed theory is not applicable, even for fast chaotic flows. As seen in Fig. 1 the approach to fast-flow limit can be non-trivial. A more detailed study shows that the nature of this approach depends on the exact nature of the evolutionary process and on the initial conditions, among other things. The current paper focuses on the fast-flow limit itself, but not on the details of how exactly this limit is approached.

**Figure 2:**
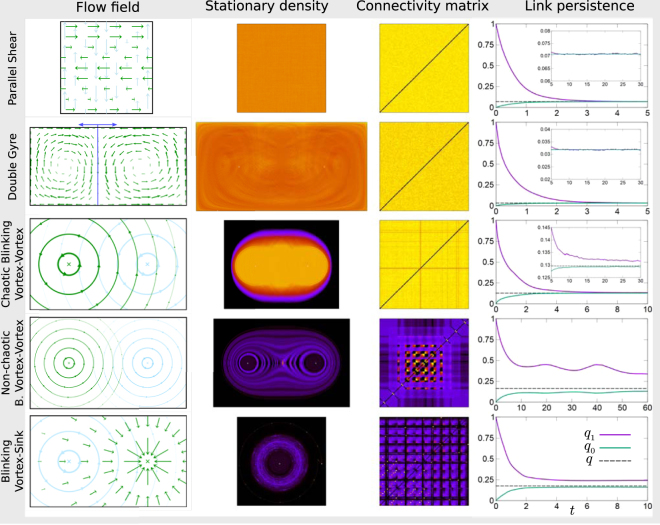
Mixing properties of different planar flows. The first column shows a graphical representation of the flow field for a selection of two-dimensional flows (see Supplemental Material). Velocity fields are periodic (modulo a random phase), and we use a period of one throughout. Green and blue arrows represent this periodic switching. The second column shows the stationary density of particles in space, as measured from simulations. The fraction of time each pair of particles spend within interaction radius from each other is shown as a connectivity matrix in the third column. Results are from simulations. The fourth column shows the measured link persistence, *q*_1_(*t*), as well as *q*_0_(*t*) and the asymptotic connectivity *q* (see text). Convergence of *q*_1_ and *q*_0_ to a common value *q* indicates that the flow mixes the system.

We next return to the commonly used verbal description of well-mixed populations, and determine whether any pair of individuals interact with the same probability. Labelling particles and tracking them as the flow proceeds we determine, for each pair, the proportion of time they are within interaction range from each other. This yields a symmetric connectivity matrix, shown in Fig. 2. Results indicate that, averaged over time, the parallel-shear flow meets the verbal criterion of good mixing; each individual is equally likely to interact with any other.

**Figure 3:**
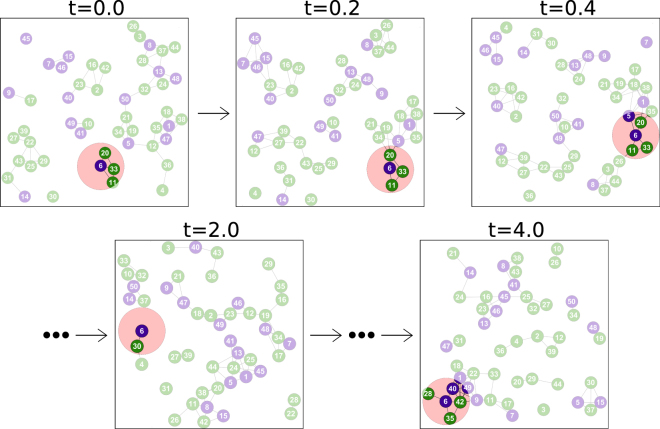
Sets of neighbours of an individual at different moments in time. The illustration shows the position of a group of particles as they are moved by the flow. We highlight the time-dependent set of neighbours of one particle. The sets of neighbours remain correlated in the frames shown in the upper row. In the lower row, however, the sets of neighbours are uncorrelated from frame to frame.

Now, consider the sequence of times at which a specific individual participates in evolutionary events, i.e., it is chosen to compete or to be replaced. In order for a system to be ‘well-mixed’ it is reasonable to require that the sets of neighbours at the time of an event are uncorrelated from those at earlier events. If this is not the case, the system has not been ‘mixed’ between the two events. We illustrate this in Fig. 3. The top row shows snapshots taken at short intervals, and demonstrates that the sets of neighbours of a particle remain correlated from one frame to the next. If evolutionary steps were to happen on these timescales the system cannot be said to be well mixed. If, on the other hand, evolutionary events occur with lower frequency, the neighbours of a particle at the time of an event are uncorrelated to those at earlier events. This is illustrated in the lower row.

To characterise this in more detail, we have measured the probability, *q*_1_(*t*), that two particles who were within interaction radius at time *t*_0_ are also connected at time *t*_0_ + *t*. In the stationary state, this is independent of *t*_0_. We refer to this quantity as the link persistence. We also measured the probability that two particles who are not connected at an earlier time are connected *t* units of time later, and denote this quantity by *q*_0_(*t*). Results are shown in Fig. 2.

We write *q* for probability that two randomly selected individuals are within interaction radius of each other, and refer to this as the connectivity. If a flow mixes the population well we expect that, eventually, the neighbours of a particle become independent of its earlier neighbours. Then *q*_0_(*t*) and *q*_1_(*t*) both tend to *q* for large enough *t*. Simulations indicate that this is the case for the parallel-shear flow (see Fig. 2). This again confirms that the flow is mixing.

The timescale *t*_*x*_ on which this regime is reached can be obtained from the simulation data shown in Fig. 2. It is the point in time when *q*_0_ and *q*_1_ have converged to their common asymptotic value, *q*. For the parallel-shear flow, as a broad order-of-magnitude estimate, we use *t*_*x*_ ≈ 5. Mixing thus occurs after approximately five periods with our choice of parameters (see Supplemental Material). The stationary particle density is uniform for this flow and, therefore, the stationary value of *q* can readily be calculated. We expect *q* = *πR*^2^/*A*, where *A* is the total area of the two-dimensional system and *R* the interaction radius. In the parallel-shear flow example shown in Fig. 2 we have *A* = 1 and *R* = 0.15, consistent with the stationary value of *q* ≈ 0.07 observed in the figure.

### Analytical description

If the typical time elapsing between evolutionary events involving a fixed particle is larger than *t*_*x*_, we can assume that the neighbours of the particle are uncorrelated to those in earlier events. This is not dissimilar to the approach of annealed random networks^36,37^. However, in our case, the interaction network is a random geometric graph^38^. Based on the assumption of uncorrelated neighbourhoods an analytical description can be constructed. In any evolutionary event, one particle is chosen at random for removal. The neighbours of this particle are obtained by randomly sampling the entire population; each particle is in the neighbourhood of the focal individual with probability *q*. Those neighbours then compete to fill the vacancy. This allows us to derive rates with which mutants replace wildtype individuals or vice-versa. From these rates we then compute the probability for a single mutant to reach fixation; for completeness we also compute times to fixation. Details of these calculations can be found in the Supplemental Material. For *r* close to one (weak selection) we find

2 $$\varphi =\frac{1-{\tilde{r}}^{-1}}{1-{\tilde{r}}^{-N}},$$

with $\tilde{r}=r+{\langle 1/k\rangle }_{c}(1-r)$, and where 〈1/*k*〉_*c*_ is the mean inverse degree among individuals who have at least one neighbour. This object depends on the connectivity *q*, which in turn depends on the interaction radius *R*. The weak-selection limit of the result for the complete graph [Eq. (1)] is recovered for *q* = 1. If the interaction radius is small, and hence the interaction graph sparse (*q* → 0), one finds 〈1/*k*〉_*c*_ ≈ 1, and $\tilde{r}=1$, i.e., neutral selection. The finite connectivity of the dynamic interaction graph acts as a suppressor of selection.

The prediction of Eq. (2) is shown in the main panel of Fig. 1 (solid black line), and agrees with simulations for small Damköhler numbers (fast flows). In the inset of the figure, we show the probability of fixation for different choices of the interaction radius *R* for fast flows. In all cases, Eq. (2) is seen to describe simulations well. The data demonstrates that, depending on the interaction radius, the fixation probability can take any value between the result for neutral selection and the one predicted by the conventional well-mixed theory.

It is important to ask how fast the flow must be for Eq. (2) to be valid. Our approach requires that each individual experiences a newly sampled set of neighbours at each event, uncorrelated from its interaction partners at earlier events. Broadly speaking, our approach applies when the typical time *τ* between events involving a particular individual is larger than the mixing time *t*_*x*_. The probability that any particular individual is involved in a given evolutionary event can be estimated as 1/*N* + (1 − 1/*N*)*q*. This means that any individual typically participates in an event every *τ* = [Da(1 + *q*(*N* − 1))]^−1^ units of time. In the example of the parallel-shear flow *q* ≈ 0.07. For a system with *N* = 100 individuals, *τ* > *t*_*x*_ when $\,{\rm{Da}}\lesssim 0.025$. If this condition is met we expect Eq. (2) to apply. This is consistent with the data in Fig. 1.

### Robustness and applicability to different flow fields

In order to test our approach further we have simulated a range of different flows, as illustrated in Fig. 2 and detailed further in the Supplemental Material. Similar to the parallel-shear flow, the double-gyre flow mixes the system at sufficiently small Damköhler numbers (uniform entries in the connectivity matrix; *q*_0_, *q*_1_ → *q*). The chaotic blinking-vortex flow is approximately mixing. The non-chaotic blinking-vortex flow and the vortex-sink flow are not mixing, as can be seen in Fig. 2. The connectivity matrix resulting from these flows indicates clusters of particles which travel the system together; the set of neighbours of any one particle can remain correlated indefinitely.

**Figure 4:**
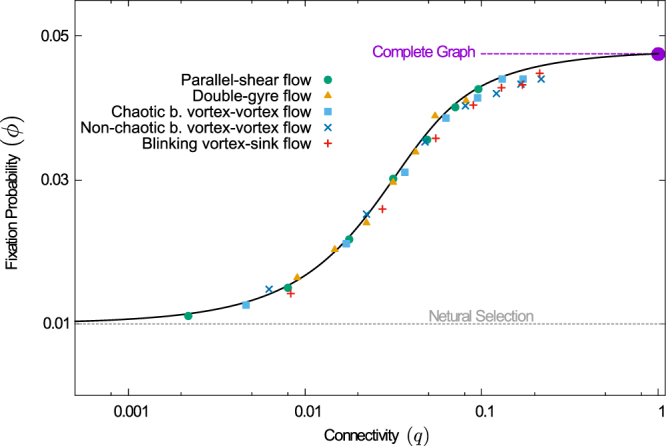
Fixation probability as a function of connectivity. Varying the interaction radius interpolates between neutral selection and the theory based on complete graphs. The fast-flow theory applies throughout, provided the flow mixes the particles well. The markers represent simulation results for different flows and different interaction radii, resulting in different connectivities, *q*. Predictions of the fast-flow theory [Eq. (2)] are shown as the solid black line. The conventional well-mixed theory [complete graph, Eq. (1)] is indicated by the filled circle at *q* = 1. The dashed gray line is for guidance only, and shows the result for neutral selection, *ϕ* = 1/*N*. (Mutant fitness *r* = 1.05, population size *N* = 100).

Results for the different flows are shown in Fig. 4. Different data points correspond to different choices of the interaction radius, resulting in different connectivities *q*. The conventional well-mixed theory is represented by the point *q* = 1; our approach interpolates between this value and the one for neutral selection in the dilute limit *q* → 0. The data in the figure demonstrates that Eq. (2) describes the fixation probability accurately for the flows that are mixing. Even for the non-chaotic blinking-vortex and vortex-sink flows it provides a good approximation.

### Dependence on the size of the population

**Figure 5:**
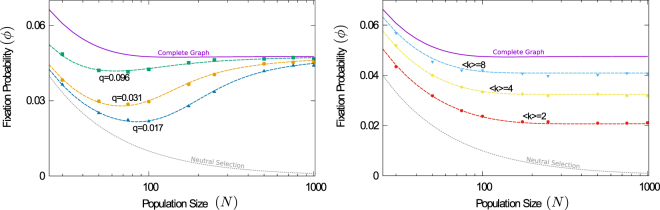
Fixation probability as a function of population size. On the left-hand panel, the interaction radius *R* is fixed as the population size is varied. This results in fixed connectivities, *q*, but the average number of neighbours of each particle increases with *N*. On the right-hand panel, the average number of neighbours, 〈*k*〉, was fixed by reducing the interaction radius as the population size increases. Markers are simulations for the parallel-shear flow. The conventional theory is shown as the thick purple line. Dashed coloured lines are the predictions of the fast-flow approach. The dashed gray line shows the result for neutral selection. (*r* = 1.05, *Da* = 0.01 in both panels).

We show the fixation probability for different population sizes in Fig. 5. In the left panel we keep the connectivity *q* fixed; the average number of neighbours of each individual is then 〈*k*〉 = (*N* − 1)*q*. Interestingly, this produces non-monotonic behaviour as a function of *N*, with minimal fixation probability at a certain population size. For small populations, the sampled neighbourhoods are so small that there is virtually no competition. The outcome of evolutionary events is dominated by the random composition of the set of neighbours of the removed individual, rather than by fitness. Effectively this results in neutral selection. In this regime, fixation of a single mutant becomes more difficult as *N* increases, and the fixation probability *ϕ* is a decreasing function of *N*. For larger populations, neighbourhoods become large enough to provide a statistically more representative sample of the entire population. Selection becomes increasingly relevant, and the fixation probability of an advantageous mutation increases. In the limit of very large populations, the neighbourhoods are a statistically accurate sample of the entire population. Therefore, the traditional well-mixed theory, based on complete graphs, is recovered. We note that 〈1/*k*〉_*c*_ tends to zero in this limit, so that $\tilde{r}=r$; the predictions of Eqs (1) and (2) then agree. In the right panel of Fig. 5 the average number of neighbours, 〈*k*〉, is kept fixed instead. Interactions are then always within local neighbourhoods, and the conventional complete-graph theory does not apply, even in large populations.

## Discussion

In the existing literature, well-mixed populations are almost always associated with complete interaction graphs. Every member of the population is connected to every other member at all times. Competition and selection in an evolutionary event then take place among all individuals. The term ‘well-mixed’ suggests that these conditions can be achieved by stirring spatial systems. As we have shown, this is often not the case. Quantitative differences between the predictions of the conventional theory and simulations of stirred populations can be observed, even when the stirring is fast and when all pairs of individuals are equally likely to interact. We have presented an alternative approach, based on the assumption that any individual, at any one time, interacts with a randomly selected subset of the population. We have demonstrated that our analytical description accurately predicts simulation results, including situations where the conventional theory does not.

**Figure 6:**
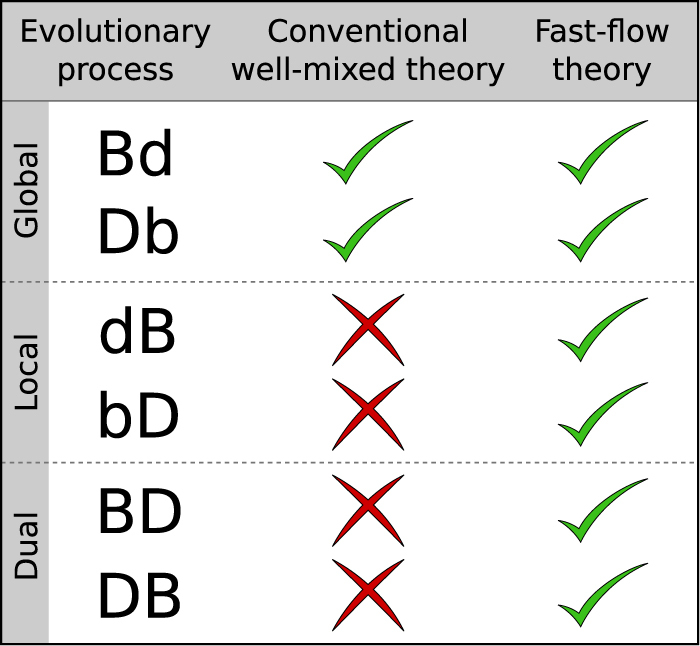
Applicability of the conventional well-mixed theory and the fast-flow theory. Evolutionary processes (see text and Supplemental Material) and indication whether the predictions of the conventional theory for well-mixed systems and of our fast-flow approach agree with simulations. Capital letters in the acronyms for the different processes indicate the presence of selection in the birth or death step. In Bd and Db competition is in the first step and therefore global. The conventional theory for well-mixed systems applies. In bD and dB competition is in the second step and therefore selection is local. In BD and DB competition takes place in both steps (dual selection). In the latter four cases the conventional theory fails. The fast-flow theory predicts simulation results in all six cases.

So far we have only discussed one type of evolutionary dynamics: a death-birth process. In the model we have described, no competition takes place when the individual for removal is determined. The reproducing individual is selected from the neighbours of the removed and proportional to fitness. This is known as ‘local selection’ and the process is referred to as a local death-birth process^3,20,39^. We write dB where the sequence of letters indicates that the death event occurs first and then the birth event, and where the capital letter indicates that selection takes place when the reproducing individual is chosen. Other variants are possible; for example, death-birth processes in which selection only acts when the individual for removal is chosen. This is known as a global death-birth process (Db). In very much the same way there are global and local birth-death processes (Bd, bD)^20,40^. Similarly selection can act at both the death and birth stages (death-birth process with dual selection (DB) or birth-death process with dual selection BD). We have tested the applicability of the conventional theory and of our approach to all six different types of processes (see Supplemental Material). We find that the conventional theory for well-mixed systems is accurate for processes in which selection only acts globally; a description based on a complete interaction graph is then appropriate. The conventional theory becomes invalid, however, when selection acts locally. As summarised in Fig. 6, the fast-flow approach we have developed applies in all cases.

If the interaction range is limited, individuals do not compete against the entire population at any one time. Our analysis shows that this limited connectivity suppresses selection. Therefore, the conventional theory for well-mixed systems overestimates the fixation probability of advantageous mutants. Our results also suggest that a disadvantageous mutant is more likely to reach fixation when it has a small interaction radius. Similar results have recently been reported by Krieger *et al*.^29^ for structured populations with mobile individuals.

The exact mechanics of evolution and the interaction range of individuals in biological or social systems are often difficult to determine. Mathematical modelling approaches frequently rely on well-mixed populations, due to the fact that these have analytical solutions. In some systems interaction may indeed occur over long distances, for example through signalling, chemical trails or the production of public goods^41–44^. Established approaches based on complete interaction graphs are then appropriate. Most systems, however, have a limited interaction range^21,45^, and as consequence conventional well-mixed theories may not apply.

There is considerable theoretical work on the effects of local interactions in static structured populations^2,14,21,45,46^. Expressions for the fixation probability of invading mutants are known. However, populations in many social or biological systems are moving and the interaction network is dynamic. The fast-flow approach provides a tool that should prove useful for the modelling of situations in which individuals are in motion.

## Electronic supplementary material

Supplemental Material
