# Supplementary material for: Stirring does not make populations well mixed
Source: Sci Rep. 2018 Mar 6;8:4068. doi: 10.1038/s41598-018-22062-w (PMC5840425; doi:10.1038/s41598-018-22062-w)
Supplement: Supplementary file 1 — Supplemental Material [file 41598_2018_22062_MOESM1_ESM.pdf]

Stirring does not make populations well mixed

Francisco Herrerías-Azcué[1], Vicente Pérez-Muñuzuri[2] and Tobias Galla[1]
francisco.herreriasazcue@manchester.ac.uk, vicente.perez@cesga.es, tobias.galla@manchester.ac.uk

[1]Theoretical Physics, School of Physics and Astronomy,
The University of Manchester, Manchester M13 9PL, United Kingdom

[2]Group of Nonlinear Physics, Faculty of Physics,
University of Santiago de Compostela E-15782 Santiago de Compostela, Spain

S1. EVOLUTIONARY PROCESSES

Two types of processes are widely used in the literature to model evolutionary dynamics: *birth-death* processes and *death-birth* processes. A birth-death process in a spatial system or network occurs in two steps: first, an individual is chosen for reproduction from the entire population; then, one of its neighbours is chosen, and is replaced by an offspring of the first individual. In the second type of process, death-birth, the individual chosen in step one is removed from the population, and one of its neighbours is chosen to produce an offspring in the 'vacant' place. (For clarity we stress again that two particles in the spatial system are considered to be 'neighbours' if their distance is less than the interaction radius.)

Each of these steps may or may not include an element of selection. Selection indicates that the choice of individual is based on fitness. Throughout our paper, we focus only on frequency-independent selection, i.e., the fitness of each type of individual only depends on the species it belongs to (mutant or wildtype), but not on the composition of its neighbourhood. In the literature, the most widely used update processes only include selection in the reproduction step. In principle, however, each individual can carry two types of fitness[1]. One is reproductive fitness; when competition occurs in the reproduction stage, it determines the probability that an individual is picked. If selection occurs during the choice of an individual for removal, we think of the resilience (against removal) as a 'death fitness'. It plays the same role as the reproduction fitness, but in the removal stage. Individuals with a higher 'death fitness' are less likely to be chosen for removal. Without loss of generality, we set both fitnesses of the wildtype to one. We write r for the mutant's reproductive fitness, and $1/d$ for its death fitness. The quantity d then describes a propensity to die.

Six possible processes can then be considered. The first three are birth-death processes. Selection can occur only in the birth step (**Bd**), only in the removal step (**bD**), or in both (**BD**). Similarly, for death-birth processes one has **Db**, **dB** and **DB**. Capital letters indicate that the step involves selection, and lower case letters indicate the absence of selection.

It is important to stress that the first individual in each birth-death or death-birth event is chosen from the *entire* population. If selection occurs at this step, this selection is *global*. The second individual is chosen among the neighbours of the first. Therefore, if selection occurs in this phase, it is *local* selection.

The most general birth-death process involves selection in both steps (**BD**, $r \neq 1$, $d \neq 1$). If $d = 1$, the birth-death process is of the type **Bd**. Selection takes place when individuals are chosen from the entire population, and it is hence global selection. For this reason, the process is also referred to as a global birth-death process. A dynamics of the type **bD** is a local birth-death process process ($r = 1$). The nomenclature for global and local death-birth processes is similar (**Db** denotes the global death-birth process, and **dB** the local death-birth process, respectively).

S2. THE LIMIT OF FAST FLOWS

S2.1. Setup and notation

A focal individual is chosen in step one of a birth-death or death-birth process. Interaction then occurs with one of its neighbours (particles within its interaction radius R). In the limit of fast flow, we assume that this neighbourhood is sampled from the entire population at random (excluding the focal individual). Each individual is in the neighbourhood with probability q, and it is not a neighbour with probability $1-q$.

The connectivity q will depend on the interaction radius. The parallel-shear flow, for example, generates a uniform stationary density of particles. Therefore, any randomly chosen individual will be a neighbour of the focal individual with probability $q = \pi R^2/A$, where A is the total area of the system. This assumes $0 < R < (A\pi)^{-1/2}$ and periodic boundary conditions. For $R \geq (A\pi)^{-1/2}$ the network is fully connected ($q = 1$).

With these assumptions the degree distribution is binomial; the probability that the focal individual has exactly k neighbours is

$$P_k = \binom{N-1}{k} q^k \left(1-q\right)^{N-1-k}. \tag{S1}$$

Assume now that there are m mutants in the population and $N-m$ wildtypes. If the focal individual is a mutant, the probability that there are l mutants among its k neighbours is

$$P^{(m)}(l|k) = \binom{k}{l} \left(\frac{m-1}{N-1}\right)^l \left(1 - \frac{m-1}{N-1}\right)^{k-l}. \tag{S2}$$

Instead, if the focal individual is a wildtype the probability that l of its k neighbours are mutants is

$$P^{(w)}(l|k) = \binom{k}{l} \left(\frac{m}{N-1}\right)^l \left(1 - \frac{m}{N-1}\right)^{k-l}. \tag{S3}$$

We next consider the situation in which k individuals compete (for example to replace a removed individual). Assuming that l of the k individuals are mutants (with fitness r) and $k-l$ are wildtypes (with fitness one) we write $g_{kl}(r)$ for the probability that a mutant is selected. We have

$$g_{kl}(r) = \frac{lr}{(k-l)+lr}. \tag{S4}$$

The quantity $1 - g_{kl}(r)$ is the probability that a wildtype is selected in this situation. If selection acts during the removal step, the probability that the individual chosen for removal is a mutant is $g_{kl}(d)$.

It is also useful to define

$$\begin{aligned} H^{(m)}(r) &= \sum_{k=1}^{N-1} \sum_{l=0}^{k} P_k\, P^{(m)}(l|k)[1 - g_{kl}(r)], \\ H^{(w)}(r) &= \sum_{k=1}^{N-1} \sum_{l=0}^{k} P_k\, P^{(w)}(l|k) g_{kl}(r), \end{aligned} \tag{S5}$$

where the double sums run over all neighbourhood compositions of the focal individual. The quantity $H^{(m)}$ describes the probability with which a wildtype is selected among the neighbours of a mutant focal individual, and $H^{(w)}$ the probability that a mutant is selected from the neighbourhood of a wildtype.

In each death-birth or birth-death event, the number of mutants in the population may increase by one ($m \to m+1$), decrease by one ($m \to m-1$), or remain unchanged. We write T_m^+ for the probability that m increases by one, and T_m^- for the probability that m decreases by one. For birth-death processes we have

$$\begin{aligned} T_m^+ &= \frac{mr}{mr+N-m} \times H^{(m)}(d), \\ T_m^- &= \frac{N-m}{mr+N-m} \times H^{(w)}(d), \end{aligned} \tag{S6}$$

where the fraction on the right-hand side in the expression for T_m^+ is the probability that the individual selected for birth is a mutant, or that it is a wildtype in the expression for T_m^-.

For death-birth processes we have

$$T_m^+ = \frac{N-m}{md+N-m} \times H^{(w)}(r),$$
$$T_m^- = \frac{md}{md+N-m} \times H^{(m)}(r). \tag{S7}$$

S2.2. Fixation probability

For any one-step process, the probability of fixation of a single mutant in a population of size N can be written as[2]

$$\phi = \frac{1}{1+\sum_{j=1}^{N-1}\prod_{m=1}^{j}\gamma_m}, \tag{S8}$$

where $\gamma_m = T_m^-/T_m^+$, sometimes known as the evolutionary drift.

For the **BD** and **DB** processes we have, respectively,

$$\gamma_m^{BD}(r,d) = \frac{N-m}{mr}\frac{H^{(w)}(d)}{H^{(m)}(d)},$$
$$\gamma_m^{DB}(r,d) = \frac{md}{N-m}\frac{H^{(m)}(r)}{H^{(w)}(r)}. \tag{S9}$$

We note that $\gamma_m^{BD}(r,d) = \left[\gamma_m^{DB}(d,r)\right]^{-1}$.

Substituting Eqs. (S9) into Eq. (S8) yields the fixation probability for the two types of processes:

$$\phi_{BD}(r,d) = \frac{1}{1+\sum_{j=1}^{N-1}\prod_{m=1}^{j}\frac{N-m}{mr}\frac{H^{(w)}(d)}{H^{(m)}(d)}} \tag{S10}$$

$$\phi_{DB}(d,r) = \frac{1}{1+\sum_{j=1}^{N-1}\prod_{m=1}^{j}\frac{md}{N-m}\frac{H^{(m)}(r)}{H^{(w)}(r)}} \tag{S11}$$

These closed-form expressions can readily be evaluated numerically.

For the global processes, there is no selection in the second step of evolutionary events, and so the expressions for $H^{(m)}$ and $H^{(w)}$ simplify considerably. If followed through, Eqs. (S10) and (S11) can be reduced to

$$\phi_{BD}(r,d=1) = \phi_{Bd} \equiv \frac{1-r^{-1}}{1-r^{-N}},$$
$$\phi_{DB}(r=1,d) = \phi_{Db} \equiv \frac{1-d}{1-d^N}, \tag{S12}$$

which are the well-known results for the complete graph[2]. It is important to note that these equations are for processes in which selection acts in the first stage of the birth-death or death-birth events. This means that selection acts globally, and that *all* N individuals in the population are in competition. The **dB** process used in the main text is slightly different. In that model, selection does not act in the first stage of each event, it acts in the second. Therefore, unless self-replacement is allowed, selection is made among $N-1$ individuals even if the interaction graph is complete (the entire population, except the individual chosen in the first stage). This leads to a slightly different expression, see e.g. Eq. (1) of the main text, even though both processes have selection only in the birth stage. As the system size increases, however, the difference between **dB** and **Bd** on a complete graph becomes small, and so the result in Eq. (1) tends to that in the first equation of (S12).

The predictions for the local birth-death and death-birth processes (**bD** and **dB**) do not reduce to expressions as simple as those in Eqs. (S12). This is perhaps to be expected from previous studies of local processes on static networks, which have shown that the traditional well-mixed theory is only valid for a very narrow set of graphs[3].

S2.3. Approximation in the limit of weak selection

We now proceed to simplify the expressions in Eqs. (S10) and (S11). We will focus on the case of the **DB** process, but similar expressions can be obtained for **BD** upon replacing d by r. We begin by simplifying the expressions for $H^{(m)}$ and $H^{(w)}$ in Eqs. (S5),

$$H^{(m)}(d) = \sum_{k=1}^{N-1} P_k \sum_{l=0}^{k} \binom{k}{l} \left(\frac{m-1}{N-1}\right)^l \left(1-\frac{m-1}{N-1}\right)^{k-l} \left(\frac{k-l}{ld+k-l}\right),$$
$$H^{(w)}(d) = \sum_{k=1}^{N-1} P_k \sum_{l=0}^{k} \binom{k}{l} \left(\frac{m}{N-1}\right)^l \left(1-\frac{m}{N-1}\right)^{k-l} \left(\frac{ld}{ld+k-l}\right). \tag{S13}$$

Since $\frac{k-l}{ld+k-l} = 1 - \frac{ld}{ld+k-l}$, we only need to compute objects of the type $\sum_{l=0}^{k} \binom{k}{l} x^l (1-x)^{k-l} \left(\frac{ld}{ld+k-l}\right)$. We expand in powers of $1-d$, and keeping only terms to first order we obtain (after re-arranging terms)

$$\begin{aligned} \frac{ld}{ld+k-l} &= 1 + \left(\frac{l}{k}-1\right) \sum_{i=0}^{\infty} \left(\frac{l}{k}(1-d)\right)^i \\ &= \frac{l}{k} d + \left(\frac{l}{k}\right)^2 (1-d) + \mathcal{O}\left((1-d)^2\right). \end{aligned} \tag{S14}$$

Using this approximation we find

$$\begin{aligned} \sum_{l=0}^{k} \binom{k}{l} x^l (1-x)^{k-l} \left(\frac{ld}{ld+k-l}\right) &= \sum_{l=0}^{k} \binom{k}{l} x^l (1-x)^{k-l} \left[d\frac{l}{k} + \left(\frac{l}{k}\right)^2 (1-d)\right] + \mathcal{O}\left((1-d)^2\right) \\ &= d\, x + (1-d)\left(\frac{x}{k} + \frac{x^2(k-1)}{k}\right) + \mathcal{O}\left((1-d)^2\right) \\ &= d\, x + (1-d)\, x^2 + \frac{(1-d)\left(x-x^2\right)}{k} + \mathcal{O}\left((1-d)^2\right). \end{aligned} \tag{S15}$$

The expressions in Eqs. (S13) can therefore be approximated as

$$\begin{aligned} H^{(m)}(d) &\approx \sum_{k=1}^{N-1} P_k \left[1 - d\left(\frac{m-1}{N-1}\right) - (1-d)\left(\frac{m-1}{N-1}\right)^2 - \frac{(1-d)\left(\left(\frac{m-1}{N-1}\right) - \left(\frac{m-1}{N-1}\right)^2\right)}{k} + \dots\right] \\ &= \left(\frac{N-m}{N-1}\right)\left[\left(\sum_{k=1}^{N-1} P_k\right) + (1-d)\left(\frac{m-1}{N-1}\right)\left(\sum_{k=1}^{N-1} P_k \frac{k-1}{k}\right)\right] + \mathcal{O}\left((1-d)^2\right) \\ &= \left(\frac{N-m}{N-1}\right) q_c \left[1 + (1-d)\left(\frac{m-1}{N-1}\right)\left(1 - \left\langle\frac{1}{k}\right\rangle_c\right)\right] + \mathcal{O}\left((1-d)^2\right), \\ H^{(w)}(d) &\approx \sum_{k=1}^{N-1} P_k \left[d\left(\frac{m}{N-1}\right) + (1-d)\left(\frac{m}{N-1}\right)^2 + \frac{(1-d)\left(\left(\frac{m}{N-1}\right) - \left(\frac{m}{N-1}\right)^2\right)}{k} + \dots\right] \\ &= \left(\frac{m}{N-1}\right)\left[\left(\sum_{k=1}^{N-1} P_k\right) - (1-d)\left(\frac{N-m-1}{N-1}\right)\left(\sum_{k=1}^{N-1} P_k \frac{k-1}{k}\right)\right] + \mathcal{O}\left((1-d)^2\right) \\ &= \left(\frac{m}{N-1}\right) q_c \left[1 + (1-d)\left(\frac{N-m-1}{N-1}\right)\left(1 - \left\langle\frac{1}{k}\right\rangle_c\right)\right] + \mathcal{O}\left((1-d)^2\right). \end{aligned} \tag{S16}$$

In these expressions we have written

$$q_c \equiv \sum_{k=1}^{N-1} P_k = 1 - P_0 \tag{S17}$$

for the probability that a randomly chosen individual has at least one neighbour, and

$$\left\langle \frac{1}{k} \right\rangle_c \equiv \frac{1}{q_c} \sum_{k=1}^{N-1} \frac{P_k}{k}. \tag{S18}$$

This expression describes the average inverse degree of all nodes with at least one neighbour.

Substituting Eqs. (S16) into Eqs. (S9), and again expanding in powers of $d-1$ and $r-1$, respectively, yields

$$\gamma^{BD}(r,d) = \frac{1}{r}\left[1-(1-d)\left(1-\langle 1/k\rangle_c\right)\right] + \mathcal{O}\left((1-d)^2\right),$$
$$\gamma^{DB}(d,r) = d\left[\frac{1}{1-(1-r)\left(1-\langle 1/k\rangle_c\right)}\right] + \mathcal{O}\left((1-r)^2\right). \tag{S19}$$

In contrast with Eqs. (S9), the first-order expressions on the right-hand side are now independent of m. It is therefore straightforward to evaluate the general expression for the fixation probability of a single mutant [Eq. (S8)]. For the **BD** process this leads to

$$\phi_{BD}(r,d) = \frac{1}{1+\sum_{j=1}^{N-1}\prod_{m=1}^{j}\frac{1}{r}\left[1-(1-d)\left(1-\langle 1/k\rangle_c\right)\right]}$$
$$= \frac{1-\left(\frac{d+\langle 1/k\rangle_c(1-d)}{r}\right)}{1-\left(\frac{d+\langle 1/k\rangle_c(1-d)}{r}\right)^N}, \tag{S20}$$

where we have neglected contributions of order $(1-d)^2$ and higher.

In the case of the global process (**Bd**, $d=1$), this reduces to Eq. (S12). For the local process (**bD**) we have $r=1$, and so Eq. (S20) reduces to

$$\phi_{BD}(r=1,d) = \phi_{bD} = \frac{1-\widetilde{d}}{1-\widetilde{d}^N}, \tag{S21}$$

with

$$\widetilde{d} = d + \langle 1/k\rangle_c (1-d). \tag{S22}$$

Similarly, for the **DB** process we have (disregarding corrections of order $(1-r)^2$ and higher)

$$\phi_{DB}(d,r) = \frac{1}{1+\sum_{j=1}^{N-1}\prod_{m=1}^{j} d\left(\frac{1}{1-(1-r)\left(1-\langle 1/k\rangle_c\right)}\right)}$$
$$= \frac{1-\left(\frac{d}{r+\langle 1/k\rangle_c(1-r)}\right)}{1-\left(\frac{d}{r+\langle 1/k\rangle_c(1-r)}\right)^N}. \tag{S23}$$

Setting $r=1$ recovers the result for the global death-birth process (**Db**) in Eq. (S12). For the local process (**dB**) we have $d=1$, and so Eq. (S23) reduces to

$$\phi_{DB}(1,r) = \phi_{dB} = \frac{1-\widetilde{r}^{-1}}{1-\widetilde{r}^{-N}}, \tag{S24}$$

with

$$\widetilde{r} = r + \langle 1/k\rangle_c (1-r). \tag{S25}$$

This corresponds to the local death-birth process (**dB**), and is the focus of the main text [see Eq. (2)].

To test the accuracy of the weak-selection approximation leading to Eq. (S24) we compare its predictions against that of the full expression of Eq. (S11) in Fig. S1. In the left-most pane, we plot the fixation probability as a function of the connectivity for different mutant fitnesses. Since the approximation requires weak-selection, deviations are expected when r is significantly different from one. In the other two panels, we show the fixation probability as a function of the population size (see also Fig. 5), keeping the connectivity q constant (central panel), or fixing the average number of neighbours $\langle k\rangle$ instead (panel on the right). As can be seen in the figure, the approximation remains valid for any system size.

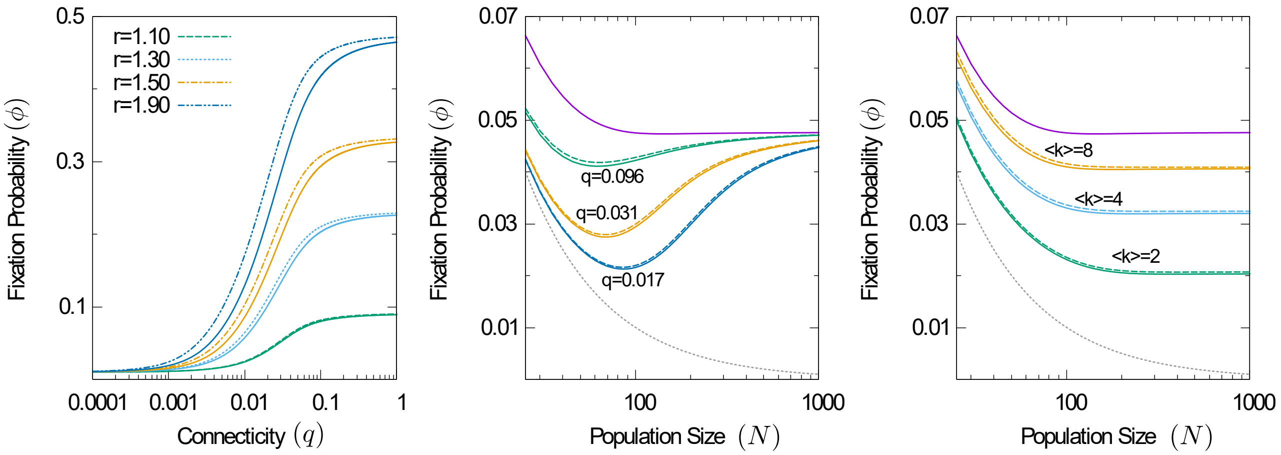

FIG. S1. **Test of the weak-selection approximation.** The left panel shows the fixation probability as a function of the connectivity q for different mutant fitnesses. The central and right panel show the fixation probability of a mutant with fitness $r = 1.05$ as a function of the population size (q fixed in central panel, average degree $\langle k \rangle$ fixed on the right). Continuous lines show the equations prior to the approximation [Eq. (S11)], and dashed lines show the equations after the approximation [Eq. (S24)]. As can be seen, the approximation is valid for all system sizes, but is sensitive at large fitnesses.

S2.4. Validity of well-mixed and fast-flow theories for the different evolutionary processes

In this section we comment on the applicability of the conventional well-mixed theory and that of our approach to the six types of birth-death and death-birth processes briefly discussed in the main text. Two individuals directly participate in each evolutionary event. The first is chosen from the entire population, and the second from the neighbours of the first individual. Two particles are 'neighbours' when the distance between them is at most R (the interaction radius). The ordering (birth-death versus death-birth) indicates whether the individual that is chosen first is removed from the population (death-birth) or whether it reproduces (birth-death). Capital letters in the acronyms indicate whether selection takes place in each of the two steps, i.e., in **BD** and **DB** processes, both steps involve selection, in **Db** and **Bd** only the first step (global selection), and in **dB** and **bD** only the second step (local selection).

S2.4.1. Global processes: Db and Bd

The global processes are obtained by setting $r = 1$ in a general death-birth process (resulting in **Db**), or setting $d = 1$ in a general birth-death process (resulting in **Bd**). Our approach for the fast-flow limit then reduces to the conventional well-mixed theory, and the predictions for the fixation probability of a single mutant are those in Eqs. (S12). These agree well with simulations, as shown in Fig. S2 (compare crosses and dark purple line). For simplicity we use $d = 1/r$; in this case the two equations in Eqs. (S12) are identical.

S2.4.2. Local processes: dB and bD

The local processes are obtained by setting $d = 1$ in a general death-birth process (resulting in **dB**), or setting $r = 1$ in a general birth-death process (resulting in **bD**). For weak selection, the predictions from our theoretical approach for the fixation probability of a single mutant is then given by Eq. (S21) for **bD**, and Eq. (S24) for **dB**. These are different from the predictions of the conventional theory for well-mixed systems, Eqs. (S12). The simulation data (empty squares and circles) in Fig. S2 agree with the predictions of Eqs. (S21, S24), shown as a dashed blue line (for $d = 1/r$ the predictions of these two equations are indistinguishable on the scale of the graph). The conventional theory (dark purple line) deviates from the simulation data.

S2.4.3. Selection in both steps: DB and BD

In the **DB** and **BD** processes selection takes place in both steps. The predictions of our approach are those of Eqs. (S20) and (S23) and are shown as a dashed red line in Fig. S2 (for $d = 1/r$ the predictions of the two equations are again indistinguishable on the scale of the figre). They agree with the simulation results (solid triangles and pentagons). The predictions of the complete-graph theory for the processes with dual selection is also shown for comparison (light purple line).

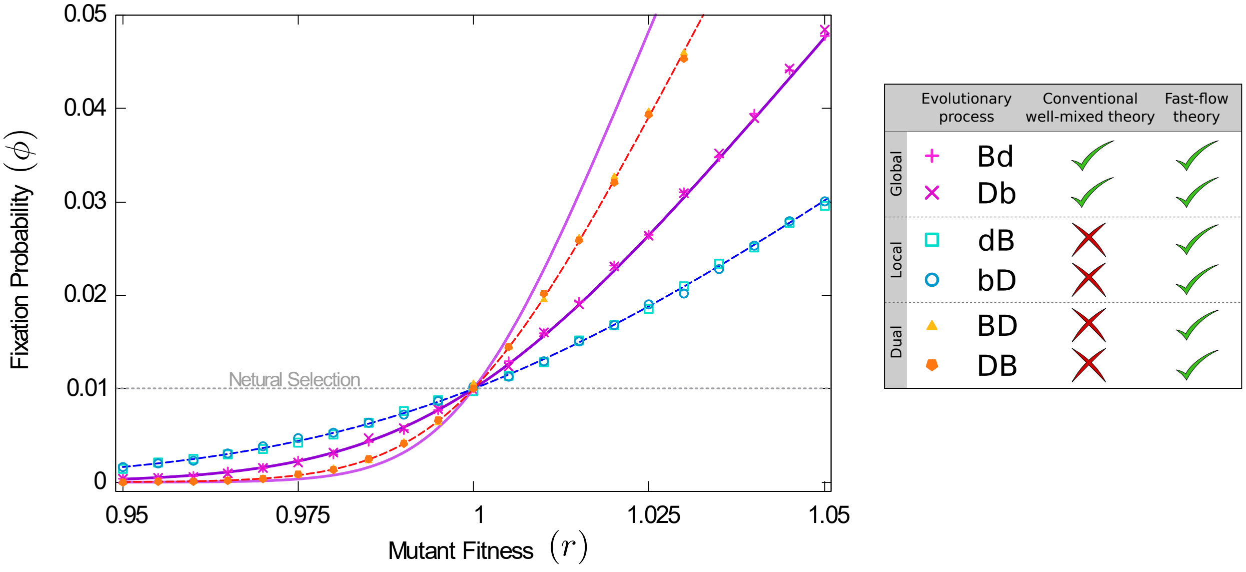

FIG. S2. **Fixation probability as a function of fitness for the different update processes.** Continuous thick lines show the conventional well-mixed theory for processes with dual selection (light purple) or selection in only one step (dark purple). Dashed lines show the fast-flow theory for dual selection (red), or local selection (blue). We use $d = 1/r$. The theoretical predictions for **BD** and **DB** are then indistinguishable on the scale of the figure, and similarly for the pairs **Bd-Db**, and **bD-dB** respectively. Simulation results are for the parallel-shear flow, with $\mathrm{Da} = 0.01$, $R = 0.1$ and $N = 100$.

S2.5. Times to fixation

Another quantity of interest is the time it takes for a single mutant to reach fixation. This is known as the conditional time to fixation, and is described in general by[4]

$$\tau = \sum_{j=1}^{N-1} \sum_{i=1}^{j} \frac{\phi_i}{T_i^+} \prod_{m=i+1}^{j} \gamma_m, \tag{S26}$$

where ϕ_i is the probability of fixation from state i, in turn given by

$$\phi_i = \frac{1 + \sum\limits_{k=1}^{i-1} \prod\limits_{l=1}^{k} \gamma_l}{1 + \sum\limits_{k=1}^{N-1} \prod\limits_{l=1}^{k} \gamma_l}. \tag{S27}$$

Using γ_m as defined in Eqs. (S9), the expression in Eq. (S26) can be written explicitly as

$$\tau_{BD} = \sum_{j=1}^{N-1} \sum_{i=1}^{j} \frac{1 + \sum\limits_{k=1}^{i-1} \prod\limits_{l=1}^{k} \frac{N-l}{lr} \frac{H^{(w)}(d)}{H^{(m)}(d)}}{1 + \sum\limits_{k=1}^{N-1} \prod\limits_{l=1}^{k} \frac{N-l}{lr} \frac{H^{(w)}(d)}{H^{(m)}(d)}} \frac{\prod_{m=i+1}^{j} \frac{N-m}{mr} \frac{H^{(w)}(d)}{H^{(m)}(d)}}{\frac{ir}{ir+N-i} \times H^{(m)}(d)},$$

$$\tau_{DB} = \sum_{j=1}^{N-1} \sum_{i=1}^{j} \frac{1 + \sum\limits_{k=1}^{i-1} \prod\limits_{l=1}^{k} \frac{ld}{N-l} \frac{H^{(m)}(r)}{H^{(w)}(r)}}{1 + \sum\limits_{k=1}^{N-1} \prod\limits_{l=1}^{k} \frac{ld}{N-l} \frac{H^{(m)}(r)}{H^{(w)}(r)}} \frac{\prod_{m=i+1}^{j} \frac{md}{N-m} \frac{H^{(m)}(r)}{H^{(w)}(r)}}{\frac{N-i}{id+N-i} \times H^{(w)}(r)}, \tag{S28}$$

with $H^{(m)}$ and $H^{(w)}$ defined as in Eqs. (S5). These equations can be readily evaluated numerically.

As with the fixation probabilities, we note that these expressions greatly simplify for the global processes. Since competition takes place among the whole population, the evolutionary drifts are simply given by $\gamma_m^{Bd}(r,1) = 1/r$ and

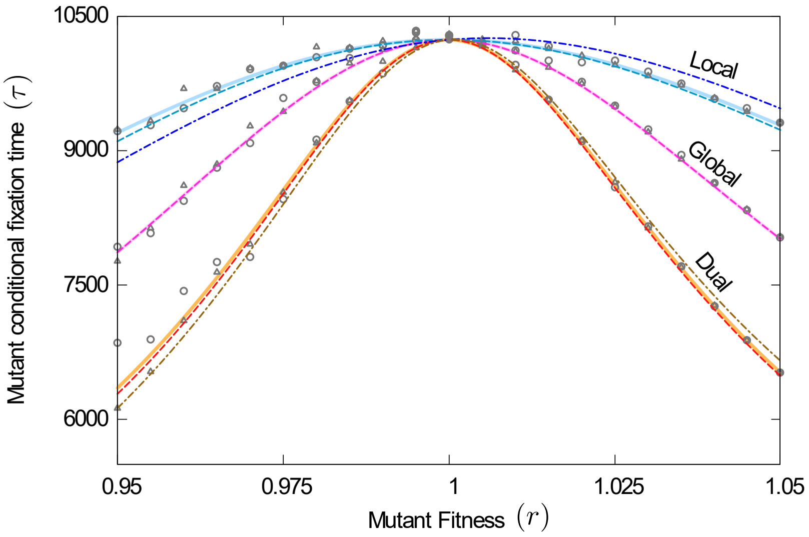

FIG. S3. **Fixation time as a function of fitness for the different update processes.** Thick continuous lines represent the fixation times prior to the approximation [Eqs. (S28)]. We use $d = 1/r$ and so db and bd processes overlap. Dashed lines show the weak selection approximation for birth-death processes, and dash-dotted lines for the death-birth processes [Eqs. (S30)]. The traditional complete-graph approach overlaps with the global processes. Simulation results are plotted with circles for birth-death processes, and triangles for death-birth processes, and were obtained using the parallel-shear flow, with $\mathrm{Da} = 0.01$, $R = 0.1$ and $N = 100$.

$\gamma_m^{Db}(1, r) = d$, which results in conditional times to fixation of

$$\tau_{Bd} = \frac{(N-1)}{q_c r\left(1 - r^{-N}\right)} \sum_{j=1}^{N-1} \sum_{i=1}^{j} \frac{(ir + N - i)\left(r^{i-j} - r^{-j}\right)}{i\,(N-i)},$$

$$\tau_{Db} = \frac{(N-1)}{q_c\left(1 - d^{N}\right)} \sum_{j=1}^{N-1} \sum_{i=1}^{j} \frac{(id + N - i)\left(d^{j-i} - d^{j}\right)}{i\,(N-i)}, \tag{S29}$$

with q_c as defined in Eq. (S17). A similar simplification is not possible for the local processes, but we can make use of the weak selection approximation obtained for the fixation probabilities.

Inserting Eqs. (S16) and (S19) into Eqs. (S28) leads to

$$\tau_{BD} = \frac{(N-1)}{q_c r\left[1 - \left(\frac{\tilde{d}}{r}\right)^{N}\right]} \sum_{j=1}^{N-1} \sum_{i=1}^{j} \frac{(ir + N - i)\left[\left(\frac{\tilde{d}}{r}\right)^{j-i} - \left(\frac{\tilde{d}}{r}\right)^{j}\right]}{i\,(N-i)\left[1 + \frac{(1-\tilde{d})(i-1)}{N-1}\right]},$$

$$\tau_{DB} = \frac{(N-1)}{q_c\left[1 - \left(\frac{d}{\tilde{r}}\right)^{N}\right]} \sum_{j=1}^{N-1} \sum_{i=1}^{j} \frac{(id + N - i)\left[\left(\frac{d}{\tilde{r}}\right)^{j-i} - \left(\frac{d}{\tilde{r}}\right)^{j}\right]}{i\,(N-i)\left[1 + \frac{(1-\tilde{r})(N-i-1)}{N-1}\right]}, \tag{S30}$$

where we used the same notation of $\tilde{r}$ and $\tilde{d}$ as in Eqs. (S25,S22). It is straightforward to see that the equations for the global processes are recovered by setting $d = 1$ (therefore $\tilde{d} = 1$) for the BD process, and $r = 1$ (therefore $\tilde{r} = 1$) for the DB process.

The predictions of Eqs. (S28) and (S30) are compared with simulation results in Fig. S3. The conditional fixation times before the approximation are shown as filled curves. Note that since we set $d = r^{-1}$, birth-death and death-birth processes are indistinguishable in the scale of the graph. The dashed lines represent the weak selection approximation. Note that $\tilde{d} = \tilde{r}^{-1} + \mathcal{O}[(r-1)^2]$, and that the last term of the denominator is different for birth-death and death-birth processes. Therefore, the two update choices do not completely overlap for local and dual selection. For the global processes the approximation recovers Eqs. (S28), as expected.

S3. DESCRIPTION OF THE FLOWS

To test our analytic predictions we have used a selection of different flows, as summarised in Fig. 2. These are all planar flows and, as a consequence, an explicit time dependence is necessary to allow for chaotic motion. Each flow is defined by a flow field, $v_x(x, y, t)$ and $v_y(x, y, t)$. We treat the individuals in the population as Lagrangian particles; their motion is governed by $\dot{x} = v_x(x, y, t)$, $\dot{y} = v_y(x, y, t)$. We write $\mathbf{v} = (v_x, v_y)$

All flows we have used are periodic $\mathbf{v}(x, y, t+T) = \mathbf{v}(x, y, t)$, where T is the period (we use $T = 1$ throughout). The only exception is the parallel-shear flow, which additionally involves a phase, randomly chosen at the beginning of each half period. Details of the flows can be found in the literature[5–9]. We briefly describe their main features below.

S3.1. Parallel-Shear

In this flow, particles move in the domain $0 \le x, y \le 1$, with periodic boundary conditions.

For the first half of the period ($nT \le t < nT + T/2$), the particles move horizontally, with a velocity which depends on their vertical position. Specifically

$$\begin{aligned} v_x(x, y, t) &= V_{\max} \sin(2\pi y_t + \phi), \\ v_y(x, y, t) &= 0. \end{aligned} \tag{S31}$$

The constant pre-factor $V_{\max}$ sets the overall magnitude of the flow. We use $V_{\max} = 1.4$. The phase ϕ is drawn randomly from the interval $[0, 2\pi)$ at the beginning of each half period.

During the second half of each period $[nT + T/2 \le t < (n+1)T]$, the particles move vertically, with a velocity that depends on their horizontal position,

$$\begin{aligned} v_x(x, y, t) &= 0, \\ v_y(x, y, t) &= V_{\max} \sin(2\pi x_t + \phi). \end{aligned} \tag{S32}$$

Within each half period the velocity of each particle is constant. In the first half of each period numerical integration can therefore be carried out using

$$x(t + \Delta t) = x(t) + \Delta t\, V_{\max} \sin[2\pi y_t + \phi], \tag{S33}$$

and in a similar way for the second half of each period.

The time step Δt does not need to be kept small, so long as the end of the half-period is not reached. In practice it is convenient to first schedule the times at which evolutionary events occur (they occur at fixed intervals, calculated based on the Damköhler number). At any one time, the time step Δt can then be chosen as the remaining time until the next evolutionary event or the end of the next half-period (whichever is shorter). This generates a very efficient numerical integration scheme.

S3.2. Double-Gyre

The spatial domain is now given by $0 \le x \le 2$ and $0 \le y \le 1$. No particular boundary conditions apply, as particles cannot leave the domain.

A *clock-wise* rotating gyre is centred on $(0.5, 0.5)$, and a *counter-clock-wise* rotating gyre on $(1.5, 0.5)$. Each gyre rotates the particles in a spiral motion. In the absence of further external driving there is no flow of particles across the line $x = 1$. However, if the transport barrier between the two gyres is driven back and forth, particles can move between the two spirals.

The flow field is given by

$$\begin{aligned} v_x(x, y, t) &= \pi M \sin\left[\pi a(t) x^2 + \pi b(t) x\right] \cos(\pi y), \\ v_y(x, y, t) &= \pi M \left[2a(t)x + b(t)\right] \cos\left[\pi a(t) x^2 + \pi b(t) x\right] \sin(\pi y), \end{aligned} \tag{S34}$$

where

$$\begin{aligned} a(t) &= u_0 \sin \frac{2\pi t}{T}, \\ b(t) &= 1 - 2u_0 \sin \frac{2\pi t}{T}. \end{aligned} \tag{S35}$$

The parameter M sets the amplitude of the flow around the gyres, and u_0 controls the movement of the barrier between the two gyres. We use $M = 1.4$ and $u_0 = 0.4$.

We simulate the flow using an Euler scheme, with time step $\Delta t = 0.001$.

S3.3. Blinking Vortex-Vortex

The blinking vortex-vortex flow describes motion around two vortices which are 'active' during alternating times. For the first half of the period the particles rotate around a vortex located at $x_c = -b$, and for the second half of the period the centre of rotation takes the position $x_c = b$. In the simulations we use $b = 0.25$.

We first describe the motion of particles around a vortex with fixed centre at $(x_c, 0)$. In this case the equations of motion are[9]

$$v_x(x,y) = -\frac{\Gamma y}{\tilde{x} + y^2}$$
$$v_y(x,y) = \frac{\Gamma \tilde{x}}{\tilde{x}^2 + y^2}, \tag{S36}$$

where $\tilde{x}(t) = x(t) - x_c$. This motion keeps the distance from the centre of the vortex constant ($r^2 \equiv \tilde{x}^2 + y^2 = \text{const}$), and results in an angular velocity $\omega = \Gamma/r^2$ which is constant for each particle, but which depends on the distance from the vortex centre. The tangential component of the velocity $\mathbf{v}$ is proportional to $1/r$. The constant Γ sets the scale of the flow velocity.

During each half-period (i.e., during rotations about a fixed vortex) the radial distance from the vortex centre remains constant. The motion is implemented conveniently through application of a rotation matrix,

$$\begin{pmatrix} \tilde{x}(t+\Delta t) \\ y(t+\Delta t) \end{pmatrix} = \begin{pmatrix} \cos(\Delta\theta) & -\sin(\Delta\theta) \\ \sin(\Delta\theta) & \cos(\Delta\theta) \end{pmatrix} \begin{pmatrix} \tilde{x}(t) \\ y(t) \end{pmatrix}, \tag{S37}$$

where

$$\Delta\theta = \frac{\Gamma}{\tilde{x}^2(t) + y^2(t)} \Delta t, \tag{S38}$$

is the rotation angle in time the interval Δt. As in the parallel-shear flow, there is no requirement to use a small time step Δt; it can be chosen as the time until the end of the next half-period or the time until the next evolutionary event (whichever comes sooner).

Depending on the choice of parameters and the initial conditions, the blinking vortex-vortex flow can either be chaotic or non-chaotic:

a. Chaotic For $\Gamma \gtrsim \Gamma_c = 0.14$ the flow is chaotic[5]. The simulations for the chaotic case shown in the main text correspond to $\Gamma \approx 0.2$.

b. Non-Chaotic If Γ is below the critical value, the flow is not chaotic. Instead domains separated by transport barriers are obtained[9]. The flow can then not be expected to be well mixing. Simulations shown in the main text correspond to $\Gamma \approx 0.01$.

Initially we place all particles in the region $-0.24 \le x, y \le 0.24$. The domain in which the particles move is in principle not bounded. However, at long times all particles in our simulations are found within a fixed bounded area.

S3.4. Blinking Vortex-Sink

This flow is very similar to the blinking vortex-vortex flow, but one of the vortices is replaced by a sink. When the sink is active particles are attracted towards its centre, and there is no angular motion. Specifically, in polar coordinates this is of the form

$$\dot{\theta} = 0,$$
$$\dot{r} = \frac{m}{2\pi r}, \tag{S39}$$

where r is the distance from the sink. This translates into $\frac{d}{dt} r^2 = m/\pi$.

The position of the particles is updated in the same way as in the blinking vortex-vortex flow for the first half of the period. When the sink is active (second half of each period), the numerical scheme involves resizing the radial distance from the sink by a factor λ. We write $\tilde{x}(t) = x(t) - x_s$, where x_s is the location of the sink. We then have

$$\begin{aligned} \tilde{x}(t+\Delta t) &= \lambda \tilde{x}(t) \\ y(t+\Delta t) &= \lambda y(t). \end{aligned} \tag{S40}$$

The scale factor is

$$\lambda = \sqrt{1 - \frac{\Delta t\, m}{\pi \left(\tilde{x}^2(t) + y^2(t)\right)}}, \tag{S41}$$

if $\Delta t\ m \leq \pi \left[\tilde{x}^2(t) + y^2(t)\right]$, and $\lambda = 0$ otherwise. The time step does not necessarily need to be small; it can be chosen in the same way as in the parallel-shear and blinking vortex-vortex flows.

The parameter m is the 'pull' strength of the sink. If m is very large, every node will be inevitably pulled to the sink during the half period in which the sink is active. The entire population will then be concentrated at $(x_s, 0)$. For sufficiently small m, more interesting dynamics are obtained. In our simulations we use $m = 0.03$ and $x_s = 0.25$.

As in the Blinking Vortex-Vortex flow, the domain in which the particles move is not bounded. However, we find that the stationary density is restricted to a finite area.

[1] Kamran Kaveh, Natalia L. Komarova, and Mohammad Kohandel, "The duality of spatial death-birth and birth-death processes and limitations of the isothermal theorem," Royal Society Open Science **2**, 140465–140465 (2015).

[2] Arne Traulsen and Christoph Hauert, "Stochastic evolutionary game dynamics," Reviews of nonlinear dynamics and complexity **II**, 25–61 (2009).

[3] Karan Pattni, Mark Broom, Jan Rychtár, and Lara J. Silvers, "Evolutionary graph theory revisited: when is an evolutionary process equivalent to the Moran process?" Proceedings of the Royal Society A: Mathematical, Physical and Engineering Science **471**, 20150334 (2015).

[4] Philipp M. Altrock and Arne Traulsen, "Fixation times in evolutionary games under weak selection," New Journal of Physics **11** (2009), 10.1088/1367-2630/11/1/013012.

[5] Julio M. Ottino, *The kinematics of mixing: stretching, chaos, and transport* (Cambridge University Press, Cambridge, UK, 1989).

[6] Pijush K. Kundu and Ira M. Kohen, *Fluid Mechanics*, 2nd ed. (Academic Press, San Diego, California, 1990).

[7] David J. Acheson, *Elementary Fluid Dynamics* (Clarendon Press, Oxford, UK, 1990).

[8] Donald F. Young, Bruce R. Munson, Theodore H. Okiishi, and Wade W. Huebsch, *A brief introduction to fluid mechanics*, 5th ed. (John Wiley & Sons, Jefferson City, 2010).

[9] Zoltán Neufeld and Emilio Hernández-García, *Chemical and biological processes in fluid flows: a dynamical systems approach* (Imperial College Press, London, UK, 2010).
